# Supplementary material for: Specific attributes of the VL domain influence both the structure and structural variability of CDR-H3 through steric effects
Source: Front Immunol. 2023 Jul 26;14:1223802. doi: 10.3389/fimmu.2023.1223802 (PMC10410447; doi:10.3389/fimmu.2023.1223802)
Supplement: Supplementary file 1 [file DataSheet_1.pdf]

Supplementary Material

1 SUPPLEMENTARY FIGURES

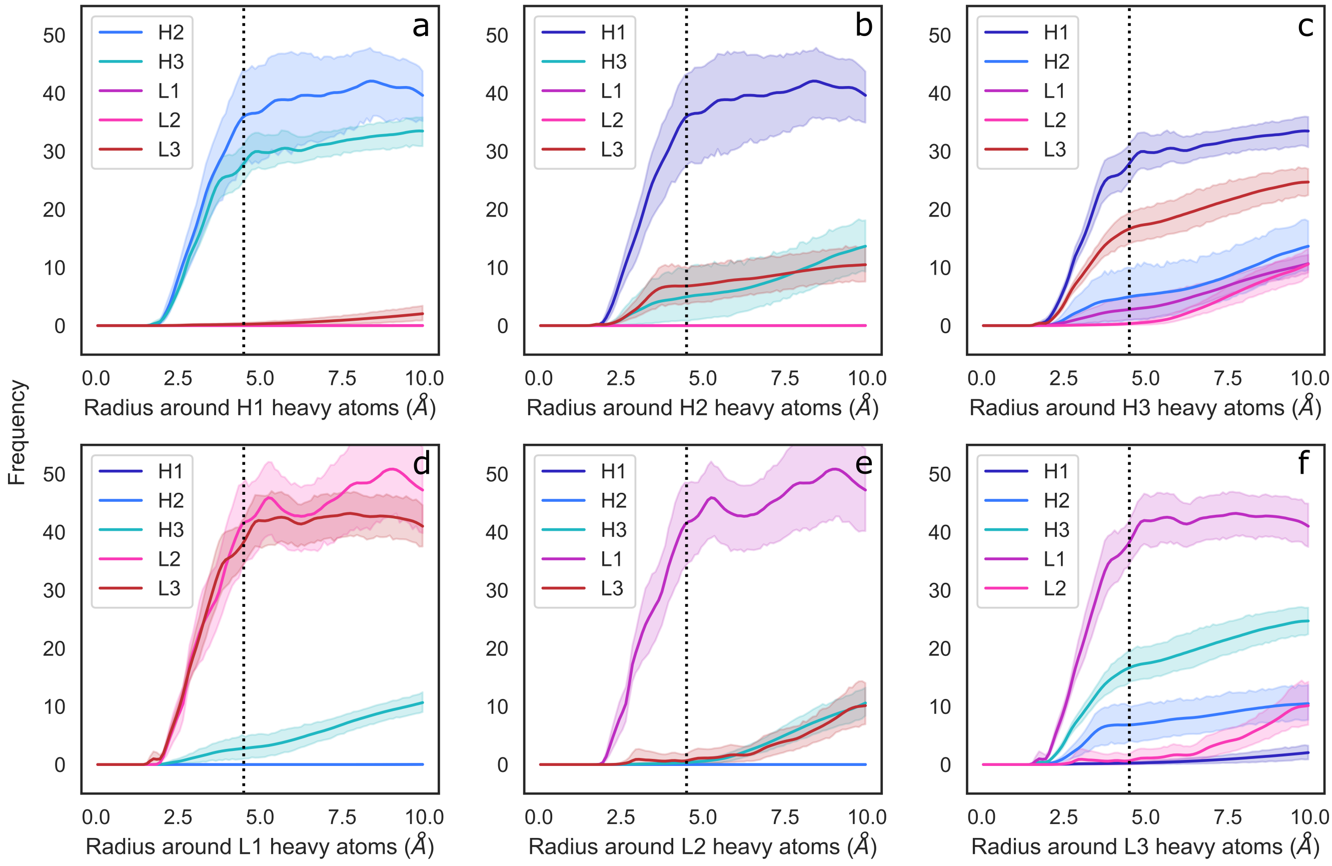

Figure S1. Radial distribution functions of CDR loops in the molecular dynamics data set, calculated RDFs using the mdtraj implementation using a bin width of 0.1Å. Shaded areas correspond to 95% confidence intervals.

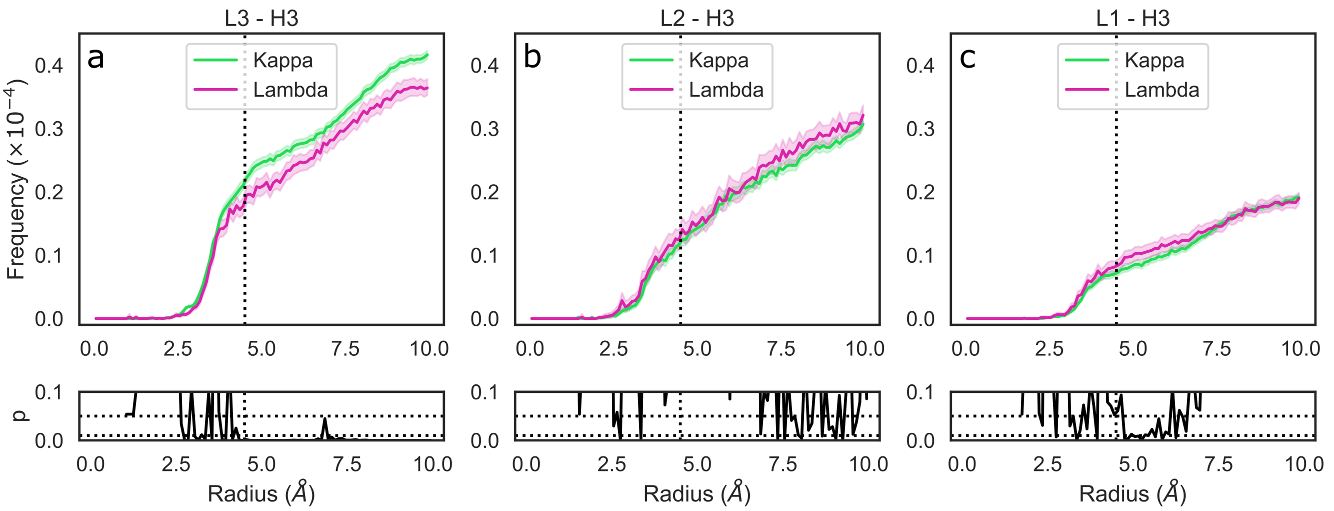

Figure S2. Radial distribution functions of CDR loops in the crystal data set separated by light chain type. e generated histograms for all pairwise CDR loop heavy atom distances using a bin width of 0.1 Å. Each bin was then normalised by dividing by the product of the volume of a spherical shell centered at 0 and located on the edges of the bin and the number of possible pairs of heavy atoms. Shaded areas are 95% confidence intervals. p-values are calculated for each bin using a t-test.
